# 

**Published:** 1913-03

**Authors:** 


*THE BRISTOL MEDICO-CHIRURGICAL SOCIETY.*

*Editor:* P. WATSON-WILLIAMS, M.D.

WITH WHOM ARE ASSOCIATED

J. MICHELL CLARKE, M.A., M.D., LL.D.,

J. LACY FIRTH, M.S., M.D., JAMES SWAIN, M.S., M.D.

J. A. NIXON, B.A., M.B., *Assistant Editor.*

*Editorial Secretary:* J. M. FORTESCUE-BRICKDALE, M.A., M.D.

LIBRARY OF CONGRESS, APR 8 1913 SMITHSONIAN DEPOSIT.

CONTENTS.

| | Page |
|---|---|
| **Robert Shingleton Smith, M.D., B.Sc., F.R.C.P.** EDITOR, 1892-1912 | 1 |
| **A Case of Acute Septicæmia due to the B. Pyocyaneus.** By J. MICHELL CLARKE, M.A., M.D., LL.D., F.R.C.P. ... ... | 4 |
| **Aleucocythæmic Leukæmia.** By RUPERT WATERHOUSE, M.D. Lond., M.R.C.P. ... ... ... ... ... ... ... ... ... | 10 |
| **Notes on a Case of Alternating Oxaluria and Phosphaturia.** By F. H. EDGEWORTH, M.D. Cantab., D.Sc. Lond. ... ... ... | 18 |
| **A Case of Alternating Oxaluria and Phosphaturia.** By C. W. J. BRASHER, M.D., Ch.B. Bristol ... ... ... ... | 21 |
| **The Intravenous Administration of Neo-Salvarsan in Syphilis.** By J. A. NIXON, M.B. Cantab., F.R.C.P. ... ... ... ... | 24 |
| **An Example of the Stokes-Adams Syndrome.** By CAREY COOMBS, M.D. Lond., M.R.C.P. .. ... ... ... ... ... ... | 30 |
| **The End-Results of Cases Operated on for Gallstones.** By A. RENDLE SHORT, M.D., B.S., B.Sc. Lond., F.R.C.S. ... ... | 34 |
| **The Treatment of Chronic Constipation: a Discussion at the Bristol Medico-Chirurgical Society, February 12th, 1913.** Introduced by F. H. EDGEWORTH, M.D. Cantab., D.Sc. Lond. ... | 40 |
| **Progress of the Medical Sciences:** | |
| **Medicine.** By J. MICHELL CLARKE, M.A., M.D., LL.D. ... ... | 48 |
| **Surgery.** By E. W. HEY GROVES, M.D., M.S. Lond. ... ... | 53 |
| **Reviews of Books** ... ... ... ... ... ... ... ... | 56 |
| **Notes on Preparations for the Sick** ... ... ... ... ... | 89 |
| **The Library of the Bristol Medico-Chirurgical Society** ... ... | 91 |
| **Meetings of Societies** ... ... ... ... ... ... ... ... | 93 |
| **Obituary Notice** ... ... ... ... ... ... ... ... ... | 94 |
| **Local Medical Notes** ... ... ... ... ... ... ... ... | 95 |

